# Primary Neurons and Differentiated NSC-34 Cells Are More Susceptible to Arginine-Rich ALS Dipeptide Repeat Protein-Associated Toxicity than Non-Differentiated NSC-34 and CHO Cells

**DOI:** 10.3390/ijms20246238

**Published:** 2019-12-11

**Authors:** Anna L. Gill, Monica Z. Wang, Beth Levine, Alan Premasiri, Fernando G. Vieira

**Affiliations:** ALS Therapy Development Institute, Cambridge, MA 02139, USA; agill2@als.net (A.L.G.); mwang@als.net (M.Z.W.); blevine@als.net (B.L.); apremasiri@als.net (A.P.)

**Keywords:** amyotrophic lateral sclerosis, *C9orf72*, ALS, dipeptide repeat proteins, arginine-rich, FTD, poly-GR, poly-PR, neurodegeneration, ALS/FTD

## Abstract

A repeat expansion mutation in the *C9orf72* gene is the most common known genetic cause of amyotrophic lateral sclerosis (ALS) and frontotemporal dementia (FTD). In this study, using multiple cell-based assay systems, we reveal both increased dipeptide repeat protein (DRP) toxicity in primary neurons and in differentiated neuronal cell lines. Using flow cytometry and confocal laser scanning microscopy of cells treated with fluorescein isothiocyanate (FITC)-labeled DRPs, we confirm that poly-glycine-arginine (GR) and poly-proline-arginine (PR) DRPs entered cells more readily than poly-glycine-proline (GP) and poly-proline-alanine (PA) DRPs. Our findings suggest that the toxicity of C9-DRPs may be influenced by properties associated with differentiated and aging motor neurons. Further, our findings provide sensitive cell-based assay systems to test phenotypic rescue ability of potential interventions.

## 1. Introduction

A hexanucleotide repeat expansion (HRE) mutation in a non-coding region of the *C9orf72* gene is currently the most common known cause of amyotrophic lateral sclerosis (ALS) and frontotemporal dementia (FTD) [1,2]. The *C9orf72* HRE (C9HRE) is the most frequent genetic association with ALS, occurring in up to 40% of familial cases and 10% of sporadic cases [3]. In ALS and FTD, having 30 or more repeats of the intronic *C9orf72* (GGGGCC)_n_ hexanucleotide is associated with disease pathology [4]. This repeat expansion of C-G-rich motifs lends itself to a phenomenon known as repeat-associated non-ATG (RAN) translation, a non-canonical form of translation associated with DNA and RNA R-loops, quadruplex structures, and hairpins [5,6,7]. In contrast to canonical translation, RAN translation occurs with no confirmed specific initiation signal or start codon and, in the case of C9HRE, does so in multiple reading frames in both sense and antisense directions [5]. This process facilitates atypical protein assembly, with resulting peptides varying in length, post-translational modification, and potentially range of function [8]. Impacts of RAN translation have been examined in many neurodegenerative diseases with repeat expansion mutations, including Huntington’s disease, numerous forms of spinocerebellar ataxia, myotonic dystrophy types 1 and 2, and spinal bulbar muscular atrophy [9,10,11,12].

There are three main hypotheses aiming to explain *C9orf72* ALS/FTD (C9ALS/FTD). The first suggests that the C9HRE is a loss-of-function mutation, resulting in pathological reduction of native *C9orf72* protein levels. While the exact function of native *C9orf72* protein is not confirmed, studies suggest that it may act as a guanine nucleotide exchange factor for small GTPases, as it has been shown to regulate endosomal trafficking and autophagy in neurons [13]. Another hypothesis suggests RNA gain-of-function neurotoxicity, resulting from RNA binding protein-sequestering RNA foci accumulating in neurons following expression of repeat expanded, intronic, *C9orf72* transcripts [14,15,16,17,18].

A third suggests that dipeptide repeat proteins (DRPs) derived from RAN translation of C9HRE RNA transcripts constitute a toxic gain-of-function mutation. These DRPs: poly-glycine-arginine (GR), poly-proline-arginine (PR), poly-glycine-proline (GP), poly-proline-alanine (PA), and poly-glycine-alanine (GA) have been shown to cause toxicity as well as interfere with vital cellular processes, including RNA biogenesis, endoplasmic reticulum function, the Notch signaling pathway, and nucleocytoplasmic transport [15,19,20,21,22,23,24,25,26]. Testing of post-mortem tissues from ALS/FTD patients has not shown a correlation between the amount or localization of C9-DRPs and neurodegenerative phenotype, which contributes to skepticism that C9-DRPs are the major contributor to C9-ALS/FTD pathogenesis [27]. However, polyGP has been detected in cerebral spinal fluid of people carrying C9HRE mutations both before and during ALS or FTD disease progression and is being explored as a biomarker of therapeutic effects in people carrying this mutation [28]. Uncovering the mechanisms of action of C9-DRPs and developing assay systems to test possible anti-DRP therapeutics remains imperative to understanding, and perhaps treating, C9ALS/FTD. Recently, multiple groups have conducted studies exploring the effects of cell-line incubation in the presence of synthesized C9-DRPs, typically between 10 and 20 repeats, and revealed signs of cytotoxicity and implicated various impaired cellular processes driving cell death [29,30,31,32,33].

In this study, we developed multiple cell-based assay systems to assess changes in cellular function and health caused by exogenous treatment with C9-DRPs. Using these assay systems, we reveal increased DRP toxicity in a neuron-like cell line, as well as in primary neurons, when compared to non-neuronal cells. Additionally, we found that among neuron-like cell populations, DRPs were more toxic to cells further differentiated toward mature neuronal phenotypes. Heightened toxicity resulting from arginine-rich DRP application to a differentiated neuron-like cell line was mirrored in testing of primary neurons. Because DRPs were applied exogenously, we accounted for differences in distribution of various DRPs into the cells. Using flow cytometry and confocal laser scanning microscopy of cells treated with fluorescein isothiocyanate (FITC)-labeled DRPs, we were able to confirm that GR and PR DRPs entered cells more readily than GP and PA DRPs, a phenomenon that has been attributed to their arginine residues [29]. Our findings suggest that the toxicity of C9-DRPs may be influenced by properties that are unique to or more prevalent in neuronal cell types. Further, our findings provide a sensitive cell-based assay system to test possible phenotypic rescue ability of potential interventions.

## 2. Results

### 2.1. Arginine-Rich DRPs Exhibit Greater Cytotoxicity in a Neuron-Like Cell Line

**Figure 1:**
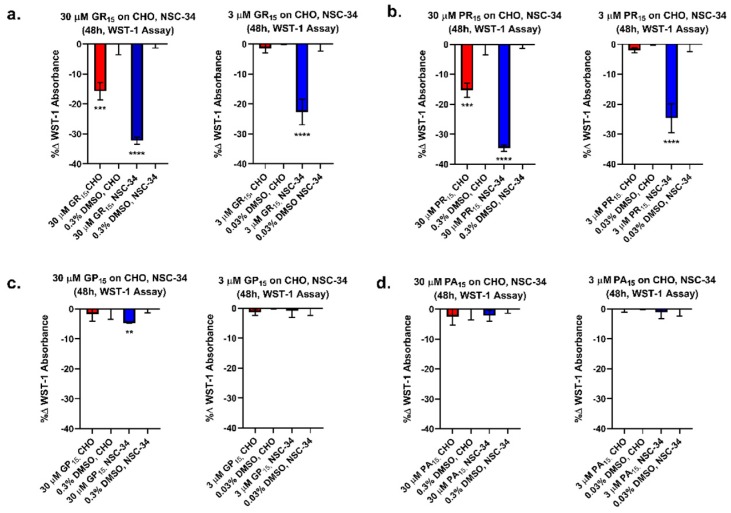
Arginine-rich dipeptide repeat proteins (DRPs) dose-dependently decrease WST-1 absorbance more potently in neuronal cell types. Dose-dependent, cell type-specific reduction of metabolic activity was observed in Chinese hamster ovary (CHO) and mouse spinal cord x neuroblastoma hybrid (NSC-34) cells treated with 30 and 3 μM doses of poly-glycine-arginine (GR_15_) (**a**) and poly-proline-arginine (PR_15_) (**b**). These results were not observed in CHO and NSC-34 cells treated with 30 and 3 μM doses of non-arginine-rich DRPs poly-glycine-proline (GP_15_) (**c**) and poly-proline-alanine (PA_15_) (**d**), with the exception of 30 μM GP_15_ on NSC-34 cells. ** denotes *p* < 0.01, *** denotes *p* < 0.001, and **** denotes *p* < 0.0001 (comparing DRP-treated groups for each cell type to corresponding Dimethyl Sulfoxide (DMSO) controls by one-way ANOVA analysis of variance followed by Dunnett’s test). Data are presented as mean values ± standard deviations (error bars).

A WST-1 tetrazolium salt-based cellular metabolic activity assay was used to compare the effects of exogenous DRP exposure across two cell lines: Chinese hamster ovary (CHO) and a mouse spinal cord x neuroblastoma hybrid cell line (NSC-34). DRP challenge resulted in decreased WST-1 absorbance in both CHO cells and NSC-34 cells. However, there was a significantly greater decrease in WST-1 absorbance in NSC-34 cells than in CHO cells (Figure 1). Specifically, 48-h incubation of CHO and NSC-34 cells with 30 and 3 µM doses of non-arginine-rich DRPs did not produce decreases in cellular metabolism compared to DMSO control with the exception of 30 µM GP_15_ in NSC-34. In contrast, 48-h incubation of CHO cells with GR_15_ and PR_15_ at 30 and 3 µM yielded decreases in metabolism of 16%, 15% (at 30 µM) and 1%, 2% (at 3 µM), respectively. Furthermore, 48-h incubation of NSC-34 cells with GR_15_ and PR_15_ at 30 and 3 µM yielded decreases in metabolism of 32%, 35% (at 30 µM) and 23%, 25% (at 3 µM), respectively. The results indicated that the neuron-like cell line NSC-34 was significantly more sensitive to arginine-rich DRP treatment when compared to the non-neuronal CHO cell line under the same experimental conditions.

### 2.2. Arginine-Rich DRPs Induce Cytotoxicity in NSC-34 in a Significant, Dose-Dependent Manner

**Figure 2:**
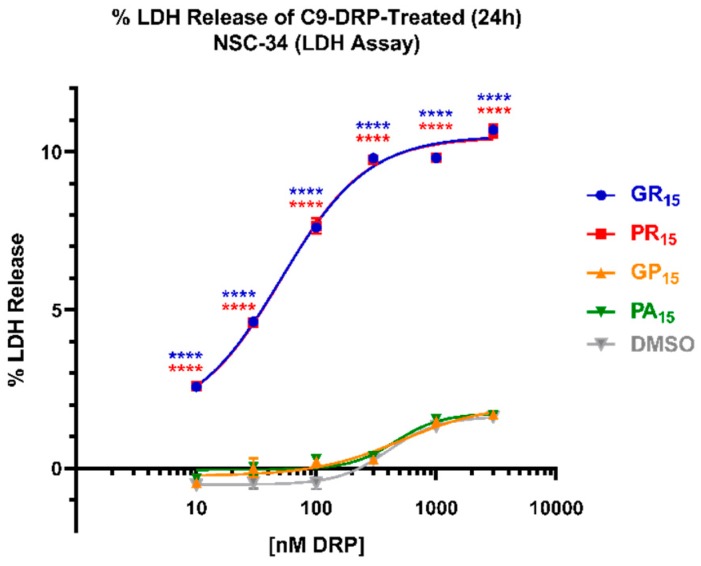
Arginine-rich dipeptide repeat proteins (DRPs) increase mouse spinal cord x neuroblastoma hybrid (NSC-34) cell LDH release in a significant, dose-dependent manner. NSC-34 cells were treated with DRPs at doses ranging from 10 nM to 3 μM for 24 h. % LDH release was calculated based on absorbance (450 nm) values detecting the release of lactate dehydrogenase (LDH) from cells, where 0% LDH release reflects a negative control of untreated NSC-34 cells, and 100% LDH release reflects a positive control of lysed NSC-34 cells. Nearly identical dose-response curves were observed in poly-glycine-arginine (GR_15_) (**blue**, EC_50_ = 50.49 ± 25.5 nM) and poly-proline-arginine (PR_15_) (**red**, EC_50_ = 55.54 ± 20.1 nM)-treated NSC-34 cells. EC_50_ values indicate arginine-rich DRPs to be at least 10 times more potent than non-arginine-rich DRPs. Dimethyl Sulfoxide (DMSO)-treated values (**gray**) represent cells that were treated with the same amount of DMSO that cells treated with DRPs dissolved in DMSO were exposed to at each dose. **** denotes *p* < 0.0001 (comparing DRP-treated values at each dose to DMSO-treated values at each dose by one-way ANOVA analysis of variance followed by Dunnett’s test). Data are presented as mean values ± standard deviations (error bars). N = 3 replicates per condition.

To further explore the effects of DRP challenge, non-differentiated/proliferating NSC-34 cells were incubated with doses ranging from 10 nM to 3 μM of PR_15_, GR_15_, PA_15_, or GP_15_ and resulting levels of cytotoxicity were measured in an LDH assay. Arginine-rich DRP treatment produced significant, dose-dependent LDH release in NSC-34 cells when incubated for 24 h, while PA_15_ and GP_15_ did not (Figure 2). Both PR_15_ and GR_15_ incubations resulted in significant LDH release as measured in this system, but neither elicited LDH release signals exceeding 10% of maximum possible signal (complete cell lysis). These levels of toxicity were achieved using PR_15_ and GR_15_ concentrations as low as 300 nM. GR_15_ and PR_15_ exhibited nearly identical dose-response profiles, with GR_15_ and PR_15_ yielding EC_50_’s of 50 ± 26 nM and 56 ± 20 nM, respectively.

### 2.3. All DRPs Induce Apoptosis in a Significant, Dose-Dependent Manner

**Figure 3:**
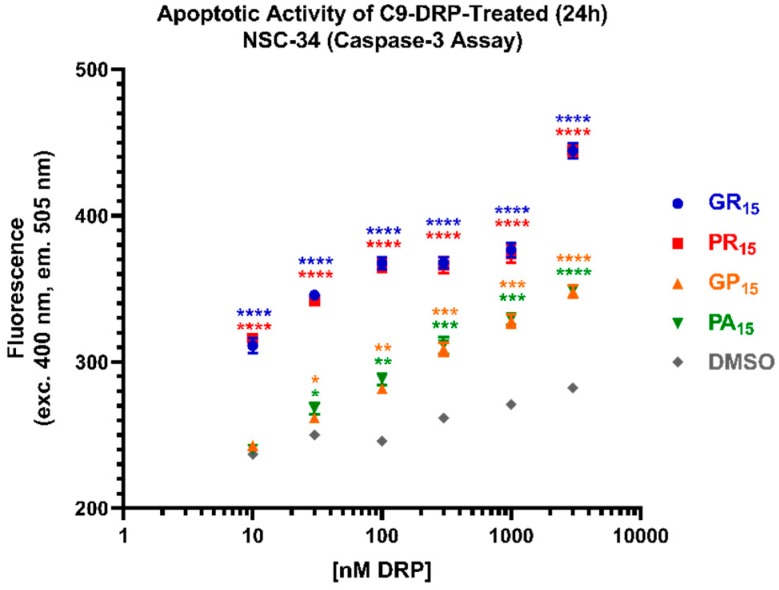
All dipeptide repeat proteins (DRPs) increase mouse spinal cord x neuroblastoma hybrid (NSC-34) cell apoptotic activity in a significant, dose-dependent manner. NSC-34 cells were treated with DRPs at doses ranging from 10 nM to 3 μM for 24 h. Higher fluorescence signal indicates more substrate cleavage by Caspase-3 enzyme activity. Identical dose-response was observed in poly-glycine-arginine (GR_15_) (**blue**) and poly-proline-arginine (PR_15_) (**red**)-treated cells, indicating significant apoptotic activity compared to Dimethyl Sulfoxide (DMSO) controls at doses as low as 10 nM. Near-identical dose-response was observed in poly-glycine-proline (GP_15_) (**orange**) and poly-proline-alanine (PA_15_) (**green**)-treated cells, with significant apoptotic activity compared to DMSO controls at doses as low as 100 nM. DMSO-treated values (**gray**) represent cells that were treated with the same amount of DMSO that cells treated with DRPs dissolved in DMSO were exposed to at each dose. * denotes *p* < 0.05, ** denotes *p* < 0.01, *** denotes *p* < 0.001, and **** denotes *p* < 0.0001 (comparing DRP-treated values to DMSO-treated values at each dose by one-way ANOVA analysis of variance followed by Dunnett’s test). Data are presented as mean values ± standard deviations (error bars). N = 3 replicates per condition.

To better characterize the cellular dysmetabolism and cytotoxicity detected using WST-1 and LDH assays, we employed an assay to detect caspase-3 activity as a surrogate for apoptotic activity. Again, cells were incubated with DRPs for 24 h. Arginine-rich DRPs, GR_15_ and PR_15,_ induced caspase-3 activity in a dose-dependent manner at all concentrations tested (Figure 3). In contrast to results from WST-1 and LDH assays, the non-arginine containing DRPs GP_15_ and PA_15_ also induced significant apoptotic activity compared to Dimethyl Sulfoxide (DMSO) controls at concentrations equal to and greater than 100 nM. While this result indicates that NSC-34 cells are sensitive to the non-arginine-rich DRPs, GP_15_ and PA_15_, it is also consistent with results indicating increased sensitivity to arginine-rich DRPs, GR_15_ and PR_15_.

### 2.4. Arginine-Rich DRPs Interfere with NSC-34 Proliferation Activity

**Figure 4:**
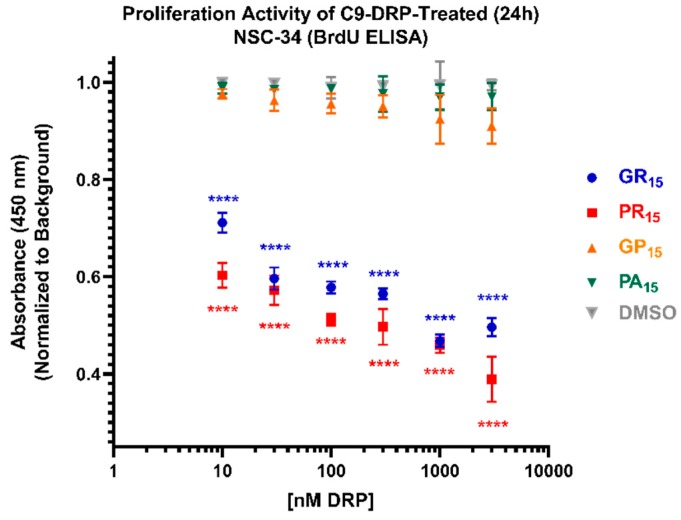
Arginine-rich dipeptide repeat proteins (DRPs) inhibit mouse spinal cord x neuroblastoma hybrid cell (NSC-34) proliferation activity in a significant, dose-dependent manner. NSC-34 cells were treated with DRPs at doses ranging from 10 nM to 3 µM for 24 h before testing. Higher absorbance values indicate higher proliferation activity as defined by the incorporation of BrdU into cell DNA. Arginine-rich DRPs poly-glycine-arginine (GR_15_) (**blue**) and poly-proline-arginine (PR_15_) (**red**) cause significant, dose-dependent proliferation inhibition, while non-arginine-rich poly-glycine-proline (GP_15_) (**orange**) and poly-proline-alanine (PA_15_) (**green**) did not. Dimethyl Sulfoxide (DMSO)-treated values (**gray**) represent cells that were treated with the same amount of DMSO that cells treated with DRPs dissolved in DMSO were exposed to at each dose. **** denotes *p* < 0.0001 (comparing DRP-treated values to DMSO-treated values at each dose by one-way ANOVA analysis of variance followed by Dunnett’s test). Data are presented as mean values ± standard deviations (error bars). N = 3 replicates per condition.

Using a BrdU ELISA, proliferation activity was the final cell viability parameter examined in actively dividing NSC-34 cells. C9-DRPs were incubated on cells for 24 h prior to readout. Non-arginine containing DRPs GP_15_ and PA_15_ did not significantly impact proliferation activity compared to DMSO controls. Arginine-rich DRPs, GR_15_ and PR_15_, both significantly suppressed proliferative activity, with PR_15_ being the strongest inhibitor (Figure 4).

### 2.5. Arginine-Rich FITC-Labeled DRP Incubation Results in Increased Labeling of NSC-34 Cells Compared to Non-Arginine-Rich DRPs

**Figure 5:**
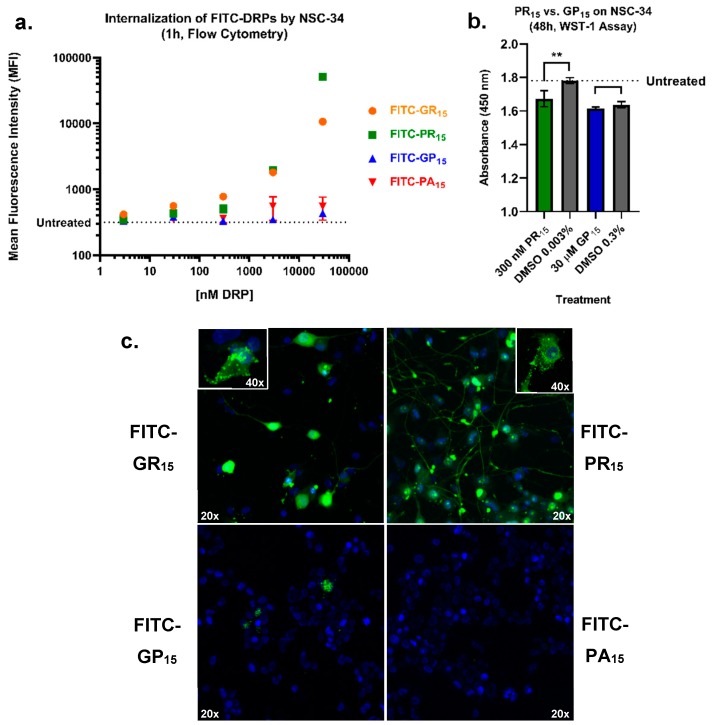
Arginine-rich fluorescein isothiocyanate-labelled dipeptide repeat proteins (FITC-DRPs) enter cells in a fast, quantifiable dose-dependent manner. Mean fluorescence intensity (MFI) values for FITC-DRP-treated mouse spinal cord x neuroblastoma hybrid cells (NSC-34) were generated from a gated region (Supplementary Figure S6) selecting only for values greater than untreated cell fluorescence values (**dotted line**). Data indicates detectable FITC-poly-glycine-arginine (GR_15_) (**orange**) and FITC-poly-proline-arginine (PR_15_) (**green**) uptake by NSC-34 at doses as low as 3 nM. Minimal entry of FITC-poly-glycine-proline (GP_15_) (**blue**) by 30 μM, and little to no entry of FITC-poly-proline-alanine (PA_15_) (**red**) was observed. Data are presented as mean values ± standard error of the mean (error bars) (**a**). Using dose-pairing of PR_15_ and GP_15_ based on flow cytometry data, a WST-1 assay indicated significant toxicity of PR_15_ (**red**) and not GP_15_ (**blue**) compared to Dimethyl Sulfoxide (DMSO) controls (**gray**). Data are presented as mean values ± standard deviations (error bars) ** denotes *p* < 0.01 (comparing DRP-dosed to corresponding DMSO control by one-way ANOVA analysis of variance followed by Dunnett’s test) (**b**). Arginine-rich FITC-DRPs localize differently in NSC-34. Confocal laser scanning microscopy of FITC-DRP treated NSC-34 reveals distinct localization of arginine-rich DRPs and limited entry of non-arginine-rich DRPs. Nuclei are 4’6-diamidino-2-phenylindole (DAPI)-stained, and FITC-DRPs appear in green. Cells were imaged using 20× and 40× objectives as indicated by white text (**c**). Additional 3-D images, as well as flow cytometry after 24 h incubation, are available as supplementary material (Supplementary Figures S1, S2, S4 and S5).

Because reconstituted exogenous DRP 15-mer constructs were used for these experiments, an assessment of cellular uptake of each DRP was conducted to determine if and to what extent the cytotoxic phenotypes revealed by each assay were influenced by cellular uptake. To test this, FITC-labelled forms of the 15-mer DRP constructs used in our cell-based assays were synthesized, and flow-cytometry was applied to assess DRP entry into NSC-34 cells over the course of 1 h (Figure 5a). Data indicated rapid and dose-dependent uptake of arginine-rich FITC-DRPs by NSC-34 cells. FITC-GP_15_ required higher concentrations to achieve similar mean fluorescence intensity as its arginine-rich counterparts. FITC-PA_15_ exhibited little uptake at concentrations tested. The data indicated one dose pairing of arginine-rich DRP PR_15_ and non-arginine-rich DRP GP_15_ where equivalent levels of cellular uptake was observed (Figure 5b). For example, NSC-34 cells likely internalized the similar levels of FITC-DRP when treated with 300 nM FITC-PR_15_ and 30 μM FITC-GP_15_. Despite similar levels of FITC signal at these concentrations, the arginine-rich DRP PR_15_ induced more robust cytotoxic phenotypes in a WST-1 assay (Figure 5b). Results in Figure 5b come with a caveat of DMSO toxicity possibly masking toxicity caused by GP_15_. Additional flow cytometry was performed in both NSC-34 and CHO cells to determine DRP levels in cells after a 24 h incubation (Supplementary Figures S1 and S2. This experiment revealed significant internalization of both FITC-GR_15_ and FITC-PR_15_ after 24-h incubation with a 3 μM dose of each. The additional experiment was performed on CHO cells to determine if differences in toxicity seen in Figure 1 resulted from differential DRP internalization across cell types. Results suggested that decreased toxicity of arginine-rich DRPs in CHO cells is not attributable to decreased DRP entry into CHO cells. Rather, more DRP uptake was observed in CHO cells, while less arginine-rich DRP toxicity was observed in that cell type. Further, an additional LDH assay was performed comparing the toxicity of FITC-labelled DRPs and non-FITC-labelled DRPs on NSC-34 cells, with no significant differences observed due to tagging DRPs with FITC (Supplementary Figure S3).

In addition, we sought to characterize DRP localization in NSC-34 cells. Using confocal laser scanning microscopy, images were captured of NSC-34 cells that had been incubated with a 3 μM FITC-DRPs for 1 h prior to fixation and mounting. Consistent with flow cytometry data, cellular uptake of arginine-rich DRPs into NSC-34 cells was more apparent than FITC-GP_15_ or FITC-PA_15_. Interestingly, the patterns of intracellular localization of GR_15_ and PR_15_ were distinct from one another (Figure 5c). One major difference is that the FITC-PR_15_ can be observed in neurites far more frequently than FITC-GR_15._ These patterns of localization are further evident in the additional three-dimensional (3-D) rotating images provided as supplements (Supplementary Figures S4 and S5).

### 2.6. NSC-34 Sensitivity to Arginine-Rich DRPs Increases with Neuronal Differentiation

**Figure 6:**
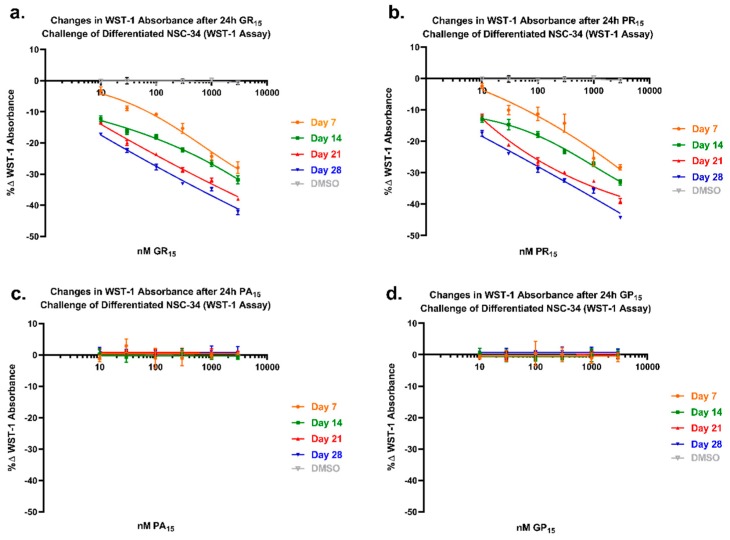
WST-1 assay of mouse spinal cord x neuroblastoma hybrid (NSC-34) cells differentiated (NSC-DIFF) for either 7, 14, 21, or 28 days revealed an increased sensitivity of NSC-DIFF to arginine-rich dipeptide repeat proteins (DRPs) with increased differentiation state. Cells treated with poly-glycine-arginine (GR_15_) or poly-proline-arginine (PR_15_) exhibited similar patterns of decreasing WST-1 absorbance with DRP treatment at each time point (**a**,**b**). Decreasing WST-1 absorbance with differentiation state was not observed in non-arginine containing DRPs poly-glycine-proline (GP_15_) and poly-proline-alanine (PA_15_) (**c**,**d**). Data are presented as average % values ± standard deviations (error bars). % values indicate the % change in toxicity DRP-treated or Dimethyl Sulfoxide (DMSO)-treated cells exhibited in comparison to untreated cells. DMSO values (gray) are calculated as an average of the four time points at each dose tested. Average value for untreated control is indicated with a dotted line. Statistical significance calculations are included in Table 1 and Table 2 below.

To examine arginine-rich DRP toxicity in relation to increased degree of neuronal differentiation, DRP-treated cells that had been differentiated for either 1, 2, 3, or 4 weeks were tested in a WST-1 assay. When allowed a longer incubation period in the neuronal differentiation protocol, a protocol widely used and well-established for inducing both physiological and morphological maturation of NSC-34 cells to a mature neuron-like phenotype [34,35,36,37], the cells became more sensitive to arginine-rich DRPs (Figure 6a,b). For example, to achieve a 25% reduction in WST-1 metabolism, cells incubated for 7 days required exposure to 1000 nM GR_15_, whereas cells incubated for 28 days only required exposure to 30 nM GR_15_. Non-arginine-rich DRP-induced toxicity did not occur or increase with neuronal differentiation state (Figure 6c,d). Further, in a different study, hydrogen peroxide (H_2_O_2_)-induced toxicity produced by either 30-min H_2_O_2_ pulse or 24 h incubation did not increase with neuronal differentiation state (Supplementary Figure S7).

### 2.7. Neonatal Mouse Derived Spinal Neurons are Most Sensitive to Arginine-Rich DRPs

**Figure 7:**
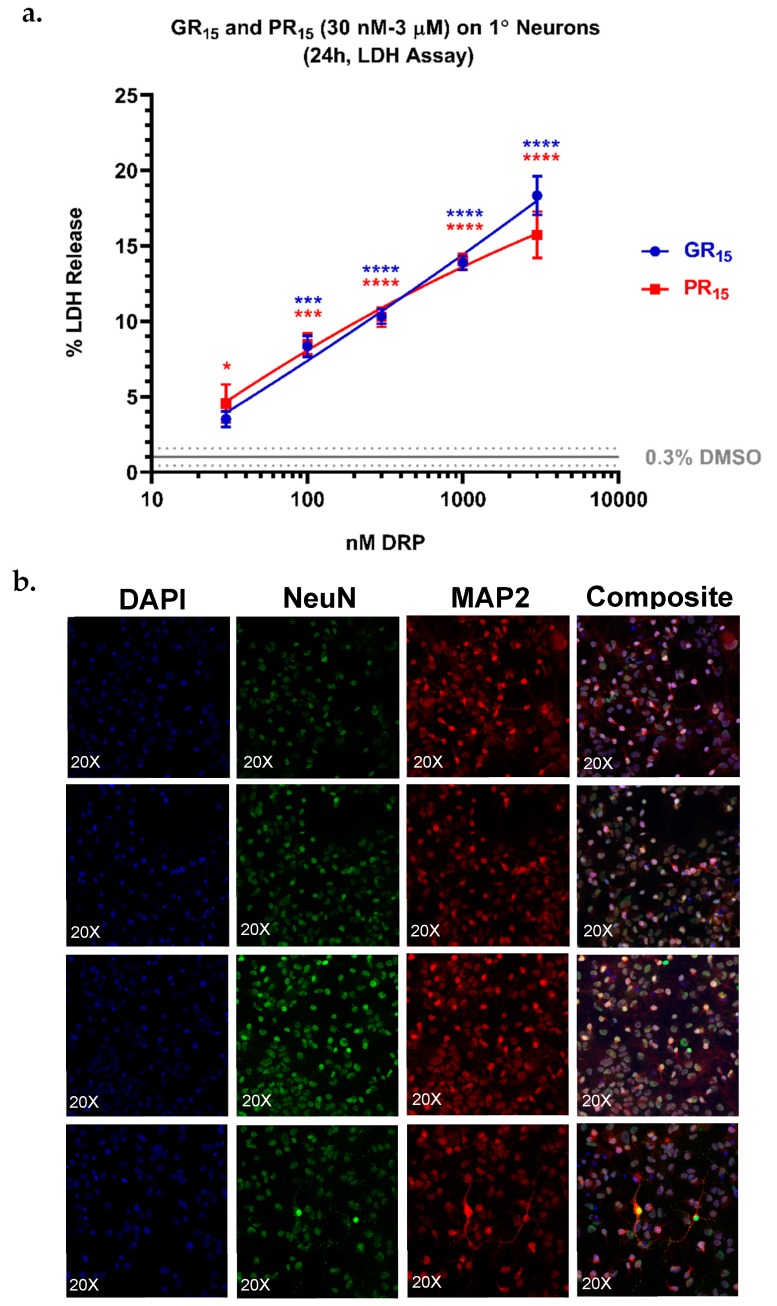
Lactate dehydrogenase (LDH) assay on primary (1°) neurons revealed an increased sensitivity to arginine-rich dipeptide repeat proteins (DRPs). 1° neurons treated with poly-glycine-arginine (GR_15_) or poly-proline-arginine (PR_15_) exhibited significant, dose-dependent LDH release, with a maximal signal of 20% LDH release at 3 µM DRP doses (**a**). Co-staining of nuclei, neuronal nuclei, and neuron cytoskeletons with 4’6-diamidino-2-phenylindole (DAPI) and antibodies against hexaribonucleotide binding protein-3 (NeuN), and microtubule-associated protein 2 (MAP2) revealed a high purity of the 1° neuron population tested (**b**). Data are presented as average % values ± standard deviations (error bars). % values indicate the % LDH release DRP-treated cells exhibited where 0% LDH release reflects a negative control of untreated 1° neurons, and 100% LDH release reflects a positive control of lysed 1° neurons. Average % LDH release induced by the highest concentration of solvent tested (0.3% DMSO) is indicated with a gray line, with gray dotted lines representing the standard deviation of this value. * denotes *p* < 0.05, *** denotes *p* < 0.001, and **** denotes *p* < 0.0001 (comparing DRP-treated values to DMSO-treated values at each dose by one-way ANOVA analysis of variance followed by Dunnett’s test). Data are presented as mean values ± standard deviations (error bars).

To more closely examine arginine-rich DRP toxicity as a function of neuron differentiation, neonatal mouse spinal neurons were isolated, cultured, and incubated with GR_15_ or PR_15_. The primary neurons were cultured for 14 days prior to being stained for neuronal markers (Figure 7b) and challenged with DRPs for 24 h. The cultured primary neurons uniformly expressed key neuronal differentiation markers (Figure 7b). Cytotoxicity was assessed by measurement of LDH release in an LDH assay. The primary neurons proved to be most sensitive to arginine-rich DRP challenge as measured by LDH release. In fact, 3 µM GR_15_ challenge produced an LDH-release signal of approximately 20% of maximum release from total cell lysis (Figure 7a).

## 3. Discussion

Generally, these results show that arginine-rich DRPs are cytotoxic. This outcome is partially consistent with multiple previous reports that arginine-rich DPRs are cytotoxic when delivered either by way of intracellular expression or through exogenous incubation [15,21,22,23,24,25,26,29,30,31,32,38]. In particular, these results echo findings that have shown that exogenous delivery of PR dipeptide repeat proteins to cells results in cytotoxicity [29,30,31,32]. Furthermore, these results are similar to previous findings suggesting that exogenous polyPR was toxic to primary rat neurons, but not to astrocytes [31].

However, our results build upon and vary from previous results in multiple ways. First of all, only one published report up to now has demonstrated that the exogenous delivery of polyGR is cytotoxic in mammalian cells and those experiments were carried out in human K562 cells, an immortalized myelogenous leukemia cell line [32]. Our experiments, carried out in non-neuronal CHO cells, in mouse motor neuron-like NSC-34 cells, and in primary neuron cells derived from neonatal mouse spinal cords, suggest increased cellular sensitivity to the arginine-rich dipeptide repeat proteins in the context of increased neuronal differentiation. Furthermore, our results demonstrate for the first time that NSC-34 cells become increasingly more sensitive to polyPR and polyGR-induced toxicity when they are further differentiated toward a mature neuronal phenotype. It is clear that this effect is a function of increased sensitivity to arginine-rich DRP specific mechanisms and not generalized increased sensitivity as a function of time because NSC-34 cells did not exhibit increased sensitivity to a control stressor, H_2_O_2_, over the course of the differentiation process. Taken a step further, these studies revealed that primary neurons were, in fact, most sensitive to polyGR and polyPR challenge. These results suggest that neuron-like cells are less resilient to polyGR and polyPR challenge than other cell types and is consistent with these DRPs playing a role in neurodegenerative conditions.

Each assay system utilized for these studies shed unique light on the possible effects of DRPs on cellular function. The WST-1 assay, a system which measures enzyme-driven tetrazolium-based salt conversion to a colored substrate as a surrogate of mitochondrial function, revealed arginine-rich-DRP-dependent impairment of cellular metabolism. Reduced, whole-well mitochondrial function could be attributable to multiple system variables: (1) reduced number of cells as a function of reduced cellular proliferation or cell death, or (2) reduced mitochondrial metabolic function of each cell, or (3) a combination of the two. The WST-1 cellular metabolic function was also inversely correlated with NSC-34 degree of neuronal differentiation, using a standard differentiation protocol widely used to produce a range of physiological and morphological changes in NSC-34 that produce a mature neuron-like phenotype [34,35,36,37]. This was not due to a general DRP-independent decline in mitochondrial function with cell aging, as DMSO-treated controls did not change in metabolic activity significantly over the same course of differentiation. Further, a control experiment assessing increased susceptibility to a control stressor, H_2_O_2_, with increased differentiation period indicated no age-related significant increase in toxicity with the exception of the highest dose tested (300 µM). These two observations do not rule out the possibility that cell age throughout the duration of differentiation is increasing sensitivity to toxicity in these experiments. However, the lack of cell age-related susceptibility observed with H_2_O_2_ application, in spite of the observed increased susceptibility to arginine-rich DRPs over the course of differentiation in the other NSC-34 experiments, is consistent with the observation that based on cells’ neuronal phenotypes or lack thereof, non-neuronal CHO cells were least sensitive to arginine-rich DRPs, while primary neurons were most sensitive to arginine-rich DRPs out of the cell lines tested, irrespective of cell aging. 

The LDH, Caspase-3, and BrdU assays were executed to shed light on the WST-1 results. The data indicate that arginine-rich DRP treatment reduced membrane integrity, increased apoptotic activity, and decreased cell proliferative activity. Interestingly, the only assay employed that indicated significant, though comparatively less robust, toxicity induced by non-arginine-rich DRPs was a caspase-3 assay, in which both GP_15_ and PA_15_ treatment led to significant caspase-3 activation. This occurred in spite of confocal microscopy and flow cytometry revealing no significant, detectable uptake of FITC-labelled, non-arginine-rich DRP species in another experiment, which could suggest that either the caspase-3 activity assay was more sensitive than the LDH and WST-1 assays or that, in comparison to LDH membrane leakage assay, that caspase-3 activity might be detectable at earlier incubation time points.

These experiments relied on cellular uptake of the various DRPs to study their effects on cellular health. Flow cytometry data at both 1 h and 24 h time points quantified relative DRP uptake, indicating significantly greater uptake of exogenous arginine-rich DRP species. While some uptake was detectable for each of the four DRPs studied, the more toxic arginine-rich DRPs were preferentially internalized by NSC-34 cells. While the increased cellular sensitivity to arginine-rich DRPs is at least in part attributable to their higher degree of cellular uptake, that difference could not completely account for the lack of toxicity of GP or PA at comparable concentrations. The only caveat of this being potential masking of GP and PA toxicity by DMSO solvent effects. Furthermore, previous reports exploring the effects of intracellular expression of poly- GR, PR, PA, and GP also indicated increased toxicity with expression of arginine-rich DRPs [15,21,22,23,24,25,26,33,38]. One limitation of this study was that while the analysis of DRP internalization was done using FITC-labelled DRP constructs, non-FITC-labelled DRPs were used in the viability assays performed. While an LDH assay comparing the toxicity of FITC-labelled DRPs to non-FITC-labelled DRPs revealed no significant differences in resulting toxicity between the two, it still cannot be absolutely certain that the uptake profiles and localization behavior of FITC- and non-FITC DRPs are the same. In addition to flow cytometry, DRP uptake was also assessed using confocal laser scanning microscopy. Careful scrutiny of the fluorescence microscopy revealed distinct localization patterns of arginine-rich DRPs FITC-GR_15_ and FITC-PR_15_. FITC-PR_15_ localized to neurites, but also was seen in the cytoplasm of cell bodies, and in nucleoli. FITC-GR_15_ could be seen faintly in some neurites, but was most evident in nuclei, and in bright punctate clusters around nuclei.

There are two notable contrasts between the findings in this report compared to previous published results. First, in the current experiments, the polyPR effect profiles closely mirrored that of polyGR. Secondly, while the toxicity of arginine-rich DRPs was repeatable and consistent across multiple assays systems, the maximum detectable effects were mild in comparison to previous reports showing near complete toxicity at some concentrations of arginine-rich DRPs. The reasons for these differences are unclear, however, there are plausible explanations. First, different cell lines and incubation times used might account for different degrees of sensitivity; indeed, the different sensitivity between CHO cells and NSC-34 cells is one of our key observations. Secondly, various groups have studied DRPs of different lengths [15,21,22,23,24,25,26,29,30,31,32,33,38] with one group providing clear evidence for changes in toxicity as a function of DRP size [30]. It is possible that results observed in our experiments with 15-mer DRP treatment on cells may not be representative of DRP modes of function at other dipeptide repeat lengths.

Overall, these experiments provide important context for previous studies exploring possible mechanisms of action driving arginine-rich DRP related toxicity. First, it has been shown that polyGR expression interferes with the Notch signaling pathway [21]. Arginine-rich DRPs have also been implicated in impaired nucleocytoplasmic transport [23,25] and impaired global mRNA translation [29]. Neurons have been shown to be differentially sensitive to disruptions of each one of these essential cellular functions and signaling pathways [39,40] suggesting that increased sensitivity of differentiated neurons could be attributable to disruptions along any one or more of the implicated processes. The results of the assays presented, including data indicating primary neurons are twice as sensitive to polyGR and polyPR challenge as compared to results in non-differentiated NSC-34 cells may serve to further implicate polyGR and polyPR interference with the above-mentioned pathways and processes. Future studies using these sensitive assay systems will explore and validate these biological pathways to determine which are more likely to be driving arginine-rich DRP-dependent neuronal toxicity. A potential narrowing of the focus of polyGR and polyPR pathology to these pathways will allow for deeper exploration and more efficient design of experiments testing therapeutic interventions against polyGR and polyPR.

## 4. Materials and Methods

### 4.1. CHO and NSC-34 Cell Culture and NSC-34 Differentiation

During proliferation, NSC-34 cells were cultured in proliferation medium consisting of DMEM/high-glucose (Millipore-Sigma, Burlington, MA, USA) supplemented with 10% fetal bovine serum (Thermo-Fisher Scientific, Cambridge, MA, USA). 1% pen strep (Thermo-Fisher Scientific, Cambridge, MA, USA), and 1% 200 mM L-glutamine solution (Thermo-Fisher Scientific, Cambridge, MA, USA). CHO cells were cultured in F-12K medium (Thermo-Fisher Scientific, Cambridge, MA, USA) supplemented with 10% fetal bovine serum (Thermo-Fisher Scientific, Cambridge, MA, USA), 1% pen strep (Thermo-Fisher Scientific, Cambridge, MA, USA), and 1% 200 mM L-glutamine solution (Thermo-Fisher Scientific, Cambridge, MA, USA). At each passage, cells were washed once with DPBS with calcium and magnesium (Thermo-Fisher Scientific, Cambridge, MA, USA) and treated for 5 min with 0.25% trypsin-EDTA (Millipore-Sigma, Burlington, MA, USA) at 37 °C, 5% CO_2_ for dissociation. For differentiation, NSC-34 cells were cultured in differentiation medium consisting of minimum essential medium Eagle/alpha modification (Millipore-Sigma, Burlington, MA, USA) supplemented with 1% fetal bovine serum (Thermo-Fisher Scientific, Cambridge, MA, USA), 1% 100× MEM non-essential amino acid solution (Millipore-Sigma, Burlington, MA, USA), 1% pen strep (Thermo-Fisher Scientific, Cambridge, MA, USA). Differentiation medium was additionally supplemented with 1 μM *trans*-retinoic acid (Millipore-Sigma, Burlington, MA, USA) at each media exchange, which occurred at plating and every 4 days. Cells were differentiated up to 4 weeks, in accordance with widely used, previously published protocol using NSC-34 cells that has been shown to produce predictable physiological and morphological outcomes [34,35,36,37]. Cells intended for use in microscopy were cultured in Biocoat poly-d-lysine/laminin 8-well culture slides (Corning Life Sciences, Tewksbury, MA, USA) until fixing and mounting steps.

### 4.2. Dipeptide Repeat Proteins for Testing

Dipeptide repeat proteins used in this study’s assays were purchased as lyophilized powders (GenicBio Limited, Kowloon, Hong Kong, China) (Table 3). Prior to solubilization with DMSO (Millipore-Sigma, Burlington, MA, USA), lyophilized protein powders were stored at −20 °C in a desiccator. FITC-proteins were stored in the dark prior to and following DMSO solubilization. Poly-glycine-alanine (GA_10_) and FITC-(GA)_10_ were also synthesized, but were not used in our assays because they could not be solubilized in DMSO.

### 4.3. WST-1 Assay

Cells plated at 3.77 × 10^4^ cells per well (CHO, NSC-34) or 1 × 10^4^ (differentiating NSC-34) in a clear, 96-well plate were challenged a day later (CHO, NSC-34) or at the desired endpoint (differentiating NSC-34) with doses of DRPs achieved by diluting 10 mM DRP from stocks in 100% DMSO to lower concentrations (specified in figures) with culture media. Controls included wells treated with equivalent DMSO concentrations to those that had been DRP-treated, and wells with untreated cells. DRPs were incubated on cells for either 24 or 48 h (specified in figures) at 37 °C, 5% CO_2_. Culture media containing DRPs was then removed and replaced with sterile phosphate-buffered saline (DPBS) with calcium and magnesium (Thermo-Fisher Scientific, Cambridge, MA, USA) supplemented with 4.5 g/L D-glucose (Millipore-Sigma, Burlington, MA, USA). One row on the top and bottom of the plate, and two columns on each side of the plate, were left without cells and contained DPBS only to minimize experimental well volume evaporation. To experimental wells containing 200 µL DPBS-glucose, 20 µL/well WST-1 reagent (Millipore-Sigma, Burlington, MA, USA) was applied and incubated at 37 °C, 5% CO_2_ until plates were read at 450 nm at 0.25, 0.5, 0.75, 1, and 1.25 h time points. Experiments included three replicates per condition. Experiments were repeated twice (executed a total of three times) including all DRP doses, and a minimum of three times using subsets of the full dose curve.

### 4.4. LDH Assay

Cells plated at 3.77 × 10^4^ cells per well in a clear, 96-well plate were challenged a day later with doses of DRPs achieved by diluting 10 mM DRP from stocks in 100% DMSO to lower concentrations (specified in figure) in culture media. Controls included wells treated with equivalent DMSO concentrations to those that had been DRP-treated, wells with untreated cells, wells with 5 µL LDH alone, wells with lysed cells, and wells with culture media only. DRPs were incubated on cells for 24 h at 37 °C, 5% CO_2_. Testing was performed using colorimetric LDH-Cytotoxicity Assay kit II (Abcam, Cambridge, MA, USA) per manufacturer’s instructions. Data analysis included calculation of % LDH release using the following equation:

$$\%\;LDH\;Release=\frac{\left(A450\;Test\;Condition-A450\;Untreated\;Control\right)}{\left(A450\;Lysed\;Control-A450\;Untreated\;Control\right)}*100\%.$$

Experiments included three replicates per condition. Experiments were repeated twice (executed a total of three times) including all DRP doses, and a minimum of three times using subsets of the full dose curve. In Figure 2, data from three technical replicates are plotted, with n = 3 referring to technical replicates. Each technical replicate comprises the average of a value tested in triplicate.

### 4.5. Caspase-3 Assay

Cells plated at 3.77 × 10^4^ cells per well were plated in a black, clear-bottom 96-well plate (Greiner Bio-One, Monroe, NC, USA) were challenged a day later with doses of DRPs achieved by diluting 10 mM DRP from stocks in 100% DMSO to lower concentrations (specified in figure) in culture media. Controls included wells treated with equivalent DMSO concentrations to those that had been DRP-treated, wells with untreated cells, wells that had been treated with wither 6 or 12 µM of the caspase-3 activator PAC-1 (Millipore-Sigma, Burlington, MA, USA), and wells with culture media alone. DRPs were incubated on cells for 24 h at 37 °C, 5% CO_2_. Testing was performed using the Caspase-3 Fluorometric Assay kit (BioVision, Milpitas, CA, USA) per manufacturer’s instructions. Experiments included three replicates per condition. Experiments were repeated twice (executed a total of three times) including all DRP doses, and a minimum of three times using subsets of the full dose curve. In Figure 3, data from three technical replicates are plotted, with n = 3 referring to technical replicates. Each technical replicate comprises the average of a value tested in triplicate.

### 4.6. BrdU ELISA

Cells plated at 3.77 × 10^4^ cells per well in a clear, 96-well plate were challenged a day later with doses of DRPs achieved by diluting 10 mM DRP from stocks in 100% DMSO to lower concentrations (specified in figure) in culture media. Controls included wells treated with equivalent DMSO concentrations to those that had been DRP-treated, wells with untreated cells that received BrdU reagent, wells with untreated cells that did not receive BrdU reagent, and wells with culture media alone. DRPs and 1× BrdU reagent were incubated on cells for 24 h at 37 °C, 5% CO_2_. Testing was performed using BrdU Colorimetric Cell Proliferation ELISA kit (Abcam, Cambridge, MA, USA) per manufacturer instructions. Experiments included three replicates per condition. Experiments were repeated twice (executed a total of three times) including all DRP doses, and a minimum of three times using subsets of the full dose curve. In Figure 4, data from three technical replicates are plotted, with n = 3 referring to technical replicates. Each technical replicate comprises the average of a value tested in triplicate.

### 4.7. Flow Cytometry

Cells plated at 6.4 × 10^5^ cells per well in 6-well plates were treated a day later with 3 µM doses of FITC-DRPs for 1 h or 24 h at 37 °C, 5% CO_2_. Cells were washed using DPBS with calcium and magnesium (Thermo-Fisher Scientific, Cambridge, MA, USA) and dissociated from plates using 0.25% trypsin EDTA (Thermo-Fisher Scientific, Cambridge, MA, USA) at 37 °C, 5% CO_2_ for 2–3 min. Dissociated cells were collected in tubes and washed twice by being spun at 1200 rpm for 5 min and resuspended in a buffer of DPBS supplemented with 1% bovine serum albumin (Jackson Immunoresearch Laboratories, West Grove, PA, USA) and 0.1% sodium azide 0.1 M solution (Millipore-Sigma, Burlington, MA, USA). Following washes, cells were run through 40 µM cell strainers (Thermo-Fisher Scientific, Cambridge, MA, USA) and loaded 250 µL/well into a clear round-bottom 96-well plate. Flow cytometry was conducted using a Guava easyCyte HT flow cytometer and guavaSoft 3.2 software (Luminex Corporation, Northbrook, IL, USA). Experiments were repeated once (executed a total of two times).

### 4.8. Immunohistochemistry and Slide Preparation for Microscopy

NSC-34 cells were plated at 1 × 10^4^ cells per well in 8-well chamber slides coated with poly-D-lysine and laminin (Corning Life Sciences, Tewksbury, MA, USA) and differentiated for 7 days, then incubated with 3 µM FITC-DRPs for 4 h at 37 °C, 5% CO_2_. Cells were then washed with DPBS with calcium and magnesium (Thermo-Fisher Scientific, Cambridge, MA, USA) three times and fixed with 4% paraformaldehyde (Electron Microscopy Sciences, Hatfield, PA, USA) for 15 min. Fixed cells were washed 3 times to remove excess paraformaldehyde, then slide chambers were removed and slides were mounted using Vectashield mounting medium with DAPI (Vector Laboratories, Burlingame, CA, USA).

Primary neurons were plated at 2.5 × 10^4^ cells per well in 8-well chamber slides coated with poly-D-lysine and laminin (Corning Life Sciences, Tewksbury, MA, USA) and cultured for 14 days. Neuronal cultures in chamber slides were then fixed at 1% paraformaldehyde in phosphate-buffered saline (PBS) for 15 min at room temperature (RT). Next, fixed cells were permeabilized in PBS with 0.1% Triton-X 100 for 5 min at RT then blocked for 1 h at RT with blocking buffer (1% BSA, 2% normal donkey serum in PBS/0.1%Triton-X). The following primary antibodies were used for immunofluorescence staining: chicken anti-MAP-2 (1:2000) (Abcam, Cambridge, MA, USA), mouse anti-Tuj1 (1:200) (Abcam, Cambridge, MA, USA), mouse anti-NeuN (Millipore, Burlington, MA, USA, 1:100), and mouse anti-SMI32 (1:100) (BioLegend, Dedham, MA, USA). The secondary antibody was biotinylated anti-chicken IgY. For visualization mouse IgG conjugated with Alexa-488 (Thermo-Fisher Scientific, Cambridge, MA, USA) or Streptavidin Alexa-594 conjugate (Thermo Fisher Scientific, Cambridge, MA, USA) were used. Slides with mounted with Vectashield mounting media with 4’, 6-diamidino-2-phenylindole (DAPI) (Vector Laboratories, Burlingame, CA, USA) for nuclei staining.

### 4.9. Confocal Laser Scanning Microscopy

Images were collected at 20× or 40× magnifications and overlaid using a Zeiss LSM 700 laser scanning microscope (Carl Zeiss Microscopy LLC, Peabody, MA, USA) and Fiji/Image J 2.0 software.

### 4.10. Isolation and Culture of Primary Neurons from Spinal Cords of Neonatal Mice

Five 0–10 day timed-pregnancy C57BL/6 mice (Jackson Laboratory, Bar Harbor, ME, USA) produced 20 pups from which spinal cords were dissected, processed, and purified to isolate primary neuron cultures according to a protocol by Eldiery et al. detailed in the Journal of Visual Experiments [41]. Briefly, solution preparation, coating of 96-well plates, spinal cord harvesting, and neuron isolation and culturing were performed using the following procedures and reagents.

HABG medium containing Hibernate A (Thermo-Fisher Scientific, Cambridge, MA, USA), 2% B27 (Thermo-Fisher), and 0.5 mM GlutaMAX (Thermo-Fisher Scientific, Cambridge, MA, USA) was prepared, sterilized, and stored at 4 °C for 24 h prior to the procedure. Neurobasal medium containing Neurobasal A (Thermo-Fisher Scientific, Cambridge, MA, USA), 2% B27 (Thermo-Fisher Scientific, Cambridge, MA, USA), 0.5 mM GlutaMAX (Thermo-Fisher Scientific, Cambridge, MA, USA), and 1% penicillin/streptomycin (Thermo-Fisher Scientific, Cambridge, MA) was prepared, sterilized, and stored at 4 °C 24 h prior to the procedure. Digestion media containing Hibernate A minus calcium (BrainBits, Springfield, IL, USA), papain (Worthington Biochemical, Lakewood, NJ, USA), and 0.5 mM GlutaMAX (Thermo-Fisher Scientific, Cambridge, MA, USA) was prepared 30 min prior to use on the day of the procedure. Four solutions, one stock tube for what would be each layer of the four-layer density gradients to be used in neuron purification, were prepared using varying volumes of OptiPrep (Millipore-Sigma, Burlington, MA, USA) and HABG on the day of the procedure, prior to assembly of density gradients. Poly-D-lysine solution containing poly-D-lysine hydrobromide (Millipore-Sigma, Burlington, MA, USA) and sterile water (Millipore-Sigma, Burlington, MA, USA) was prepared in advance of the procedure and stored at −20 °C until use. Laminin solution containing 1 mg/mL mouse laminin (Thermo-Fisher Scientific, Cambridge, MA, USA) and neurobasal medium (Thermo-Fisher Scientific, Cambridge, MA, USA) was prepared on the day of the procedure. Siliconized, flame-polished Pasteur pipettes were prepared in the days prior to the procedure using a 1:20 solution of Dichlorodimethylsilane (Millipore-Sigma, Burlington, MA, USA) in chloroform (Millipore-Sigma, Burlington, MA, USA) overnight, and 9” glass Pasteur pipettes (Millipore-Sigma, Burlington, MA, USA) and were autoclave-sterilized on the day of the procedure.

On the day prior to the procedure, sterile 96-well plates were coated with poly-D-lysine (PDL) solution and left in a laminar flow hood overnight. On the day of the procedure, PDL was removed from the wells and wells were washed with sterile water (Millipore-Sigma, Burlington, MA, USA) for 5 min before water was removed from wells and plates were left in hood to dry for 1 h. Once dry, plates were coated with laminin solution and left for 2 h at room temperature (RT), then aspirated prior to neuron plating.

For spinal cord harvesting, all instruments were autoclave-sterilized prior to procedure. Three-day-old pups were placed in an isoflurane chamber until cessation of movement was confirmed using a leg pinch. Heads were separated from the bodies using scissors with pups in the prone position. While stabilizing hind legs and arms on the procedure table, with dorsal sides facing the user, skin was removed using curved iris scissors and spinal columns were cut from first the lumbar region and then the body. Spinal columns were washed sequentially in petri dishes containing sterile PBS (Thermo-Fisher Scientific, Cambridge, MA, USA) to remove excess tissue. A 22G needle and syringe filled with sterile PBS was inserted into the caudal end of each spinal column and flushed cranially to allow the spinal cords to be removed and placed in a petri dish. Spinal cords were then transferred to 15 mL sterile tubes containing 5 mL HABG on ice.

For neuron isolation from the harvested spinal cords, first tissue was minced by gently shaking tubes before transferring tissue to a petri dish and dicing with a sterile razor blade to form roughly 0.5 mm pieces. Minced tissue was transferred into a tube containing HABG and placed in a 30 °C water bath for 30 min, with gentle shaking intermittently. During this incubation, density gradients were prepared in sterile tubes by carefully layering the four density gradient solutions, once for each cord isolated. Tissues were then removed from water bath, and HABG was removed and replaced with sterile 37 °C digestion medium. Tissues in digestion medium were incubated in a 30 °C with gentle shaking intermittently for 30 min. Digestion medium was then removed and trituration was performed 10× for 45 s for each tube using sterile, siliconized Pasteur pipettes, after which 2 mL of each sample was moved to a collection tube and this process was repeated until the collection tube contained 6 mL. Collection tube contents were then transferred to density gradients, and centrifuged for 15 min at 800× *g*, 22 °C.

Layer 3 of each density gradient, containing the most pure neuron population, was removed from each tube and pooled to one final collection tube. Residual density gradient was diluted out by adding HABG to the final collection tube. Final collection tube was centrifuged at 200 × *g* for 2 min at 22 °C. Supernatant was removed, cells were suspended, and HABG addition and centrifugation was repeated per the previous step. Supernatant was once again removed, and pellet was resuspended in neurobasal medium. The sample was the counted and plated at a density of 1.3 × 10^4^ cells per well in the PDL/laminin-coated 96-well plates prepared prior, as well as at 2.5 × 10^4^ c/w in chamber slides pre-coated with PDL/laminin (Corning Life Sciences, Tewksbury, MA, USA).

Once plated, primary neurons were cultured for 14 days prior to testing/staining in neurobasal medium (prepared fresh at each use per paragraph 1) supplemented with the following growth factors: 10 ng/mL IGF-1 (R&D Systems, Minneapolis, MN, USA), 25 ng/mL CTNF (Peprotech, Rocky Hill, NJ, USA), 10 ng/mL NT3 (Peprotech, Rocky Hill, NJ, USA), and 10 ng/mL BDNF (R&D Systems, Minneapolis, MN, USA). Media was changed every 3 days, leaving ¼ total volume conditioned media in each well during media changes. Cells that were plated in 96-well plates were treated with DRPs and controls in triplicate, according to LDH assay protocol specified in Section 4.4 of these methods. Cells that were plated in chamber slides were stained for purity according to immunohistochemistry protocol specified in Section 4.8 of these methods.

## Figures and Tables

**Table 1:** Statistical testing of data in Figure 6 indicates significant differences in mouse spinal cord x neuroblastoma hybrid (NSC-34) cell sensitivity to arginine-rich dipeptide repeat proteins (DRPs) with increasing differentiation ^1^.

DRP	Endpoint	3 µM	1 µM	300 nM	100 nM	30 nM	10 nM
	Day 14	****	ns	****	****	****	****
GR_15_	Day 21	****	****	****	****	****	****
	Day 28	****	****	****	****	****	****
	Day 14	**	ns	****	****	***	****
PR_15_	Day 21	****	****	****	****	****	****
	Day 28	****	****	****	****	****	****
	Day 14	ns	ns	ns	ns	***	ns
PA_15_	Day 21	ns	ns	ns	ns	**	ns
	Day 28	ns	ns	ns	ns	****	ns
	Day 14	ns	ns	ns	*	ns	ns
GP_15_	Day 21	ns	ns	ns	ns	ns	ns
	Day 28	ns	*	ns	*	ns	ns

^1^ * denotes *p* < 0.05, ** denotes *p* < 0.01, *** denotes *p* < 0.001, and **** denotes *p* < 0.0001 comparing changes in WST-1 absorbance at each differentiation endpoint (day 14, day 21, day 28) to values at day 7 endpoint by two-way ANOVA analysis of variance followed by Dunnett’s test.

**Table 2:** Additional statistical testing of data in Figure 6 further indicates significant differences in mouse spinal cord x neuroblastoma hybrid (NSC-34) cell sensitivity to arginine-rich dipeptide repeat proteins (DRPs) with increasing differentiation ^1^.

DRP	Comparison	3 µM	1 µM	300 nM	100 nM	30 nM	10 nM
	D7 vs. D14	**	ns	****	****	****	****
GR_15_	D14 vs. D21	****	****	****	****	****	ns
	D21 vs. D28	****	****	****	****	****	***
	D7 vs. D14	**	ns	****	****	***	****
PR_15_	D14 vs. D21	****	****	****	****	****	ns
	D21 vs. D28	****	**	ns	ns	***	****
	D7 vs. D14	ns	ns	ns	ns	***	ns
PA_15_	D14 vs. D21	ns	ns	ns	ns	ns	ns
	D21 vs. D28	ns	ns	ns	ns	ns	ns
	D7 vs. D14	ns	ns	ns	*	ns	ns
GP_15_	D14 vs. D21	ns	ns	ns	ns	ns	ns
	D21 vs. D28	ns	ns	ns	ns	ns	ns

^1^ * denotes *p* < 0.05, ** denotes *p* < 0.01, *** denotes *p* < 0.001, and **** denotes *p* < 0.0001 comparing changes in WST-1 absorbance between groups specified in “comparison” column by two-way ANOVA analysis of variance followed by Tukey’s multiple comparisons test. “D” in comparison column denotes “Day,” as in days differentiated.

**Table 3:** Details of DRP construct properties and storage.

Supplier	Sequence	Purity (%)	Molecular Weight (g)	Solubilized in	[Stock]	Stored at (°C)
GenicBio	(GR)15	94.53	3216.57	Sterile DMSO	10 mM	4
GenicBio	(PR)15	93.17	3817.53	Sterile DMSO	10 mM	4
GenicBio	(GP)15	94.49	2330.56	Sterile DMSO	10 mM	4
GenicBio	(PA)15	90.75	2540.91	Sterile DMSO	10 mM	4
GenicBio	FITC-(GR)15	92.15	3719.11	Sterile DMSO	10 mM	4
GenicBio	FITC-(PR)15	94.52	4320.07	Sterile DMSO	10 mM	4
GenicBio	FITC-(GP)15	95.50	2833.05	Sterile DMSO	10 mM	4
GenicBio	FITC-(PA)15	94.69	3043.44	Sterile DMSO	10 mM	4

## Supplementary Materials

Supplementary materials can be found at https://www.mdpi.com/1422-0067/20/24/6238/s1.

## Abbreviations

DRP	Dipeptide Repeat Protein
HRE	Hexanucleotide Repeat Expansion
C9HRE	C9orf72 Hexanucleotide Repeat Expansion Mutation
RAN	Repeat-Associated non-ATG
ALS	Amyotrophic Lateral Sclerosis
FTD	Frontotemporal Dementia
C9ALS/FTD	C9orf72 Amyotrophic Lateral Sclerosis/Frontotemporal Dementia
CHO	Chinese Hamster Ovary cell line
NSC-34	Mouse Spinal Cord x Neuroblastoma Hybrid cell line
