# Supplementary material for: Primary Neurons and Differentiated NSC-34 Cells Are More Susceptible to Arginine-Rich ALS Dipeptide Repeat Protein-Associated Toxicity than Non-Differentiated NSC-34 and CHO Cells
Source: Int J Mol Sci. 2019 Dec 11;20(24):6238. doi: 10.3390/ijms20246238 (PMC6941034; doi:10.3390/ijms20246238)
Supplement: Supplementary file 1 [file ijms-20-06238-s001.zip › ALG_supplement_ijms-632997 for proof.pdf]

Supplementary Materials:

Contents:

- **Supplementary Figure S1:** Flow cytometry of CHO cells treated with FITC-DRPs for 24h indicates that DRPs remain in CHO cells after 24h.

- **Supplementary Figure S2:** Flow cytometry of NSC-34 cells treated with FITC-DRPs for 24h indicates that DRPs remain in NSC-34 cells after 24h.

- **Supplementary Figure S3:** LDH assay on NSC-34 cells treated with a dose range of untagged (NKD) or FITC-labelled DRPs for 24h indicates no appreciable differences in toxicity.

- **Supplementary Figure S4:** 3-D rotating image of 1h, 3 μM FITC-GR_{15}-treated NSC-34 (composite of blue DAPI and green FITC-GR_{15} channels) indicate GR_{15} internalization.

    a. **Supplementary Figure S4a:** 3-D rotating image of 1h, 3 μM FITC-GR_{15}-treated NSC-34 (blue DAPI channel only).

    b. **Supplementary Figure S4b:** 3-D rotating image of 1h, 3 μM FITC-GR_{15}-treated NSC-34 (green FITC-GR_{15} channel only).

- **Supplementary Figure S5:** 3-D rotating images of 1h, 3 μM FITC-PR_{15}-treated NSC-34 (composite of blue DAPI and green FITC-PR15 channels) indicate PR_{15} internalization.

- **Supplementary Figure S6:** Histograms and forward versus side-scatter plots for flow cytometry data presented in Figure 5 of the text.

- **Supplementary Figure S7:** WST-1 assay on 7, 14, 21, and 28-day differentiated NSC-34 cells treated with either a 30-minute pulse of hydrogen peroxide followed by 24h incubation, or 24h incubation with hydrogen peroxide revealed no distinct differences in sensitivity based on differentiation state with the exception of the highest dose tested (300 μM).

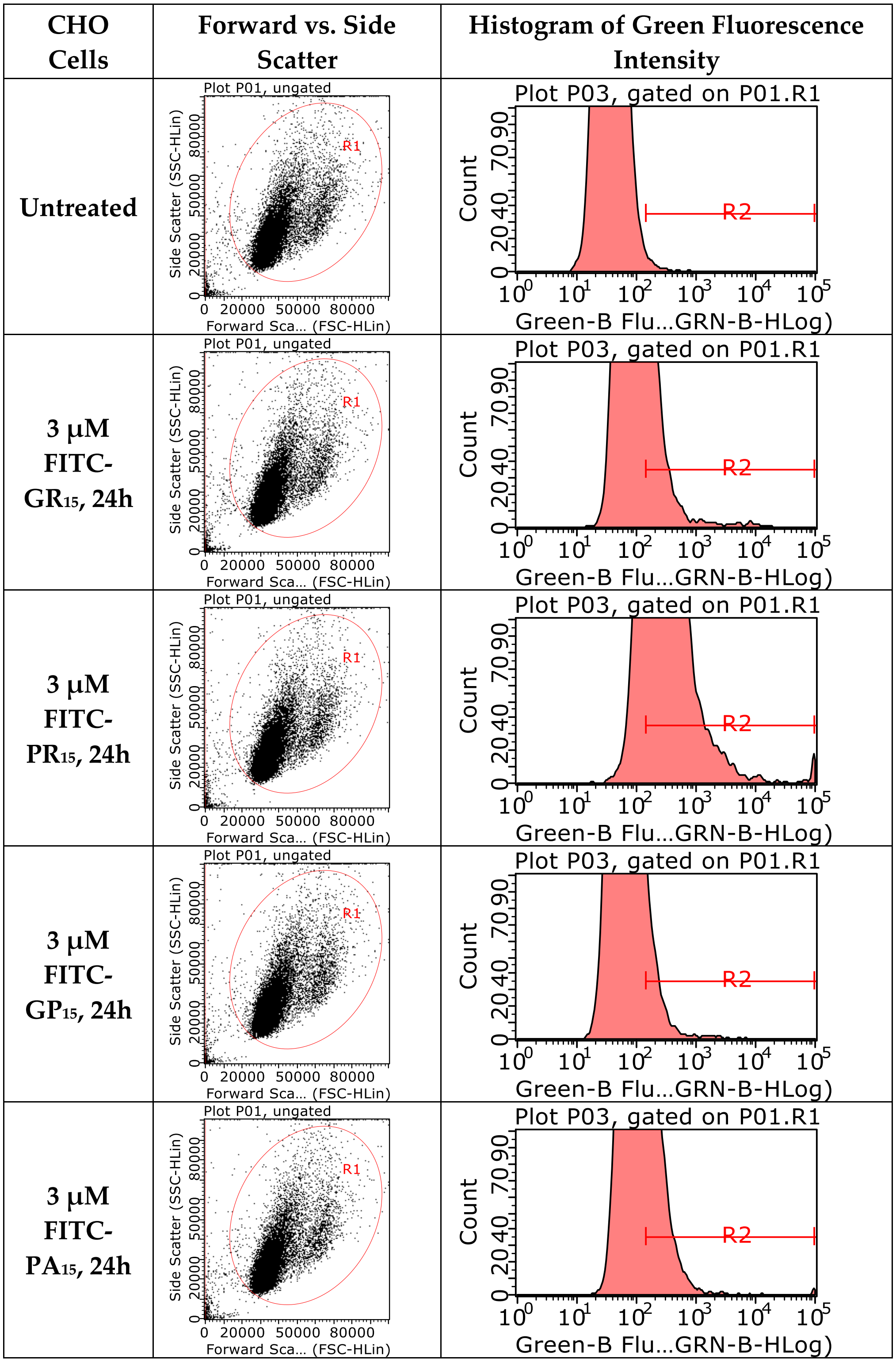

Supplementary Figure S1. Arginine-rich fluorescein isothiocyanate-labelled dipeptide repeat proteins (FITC-DRPs) remain in Chinese hamster ovary (CHO) cells after 24h incubation. Treatment conditions (Left), forward versus side scatter (Middle) and histograms of mean green fluorescence intensity (Right) indicate FITC-DRP internalization and remainder in CHO cells over 24h, especially in the case of arginine-rich FITC-DRPs. Forward versus side scatter are gated to exclude cellular debris. Histograms are gated such that region R2 includes all green fluorescence signals more positive than untreated.

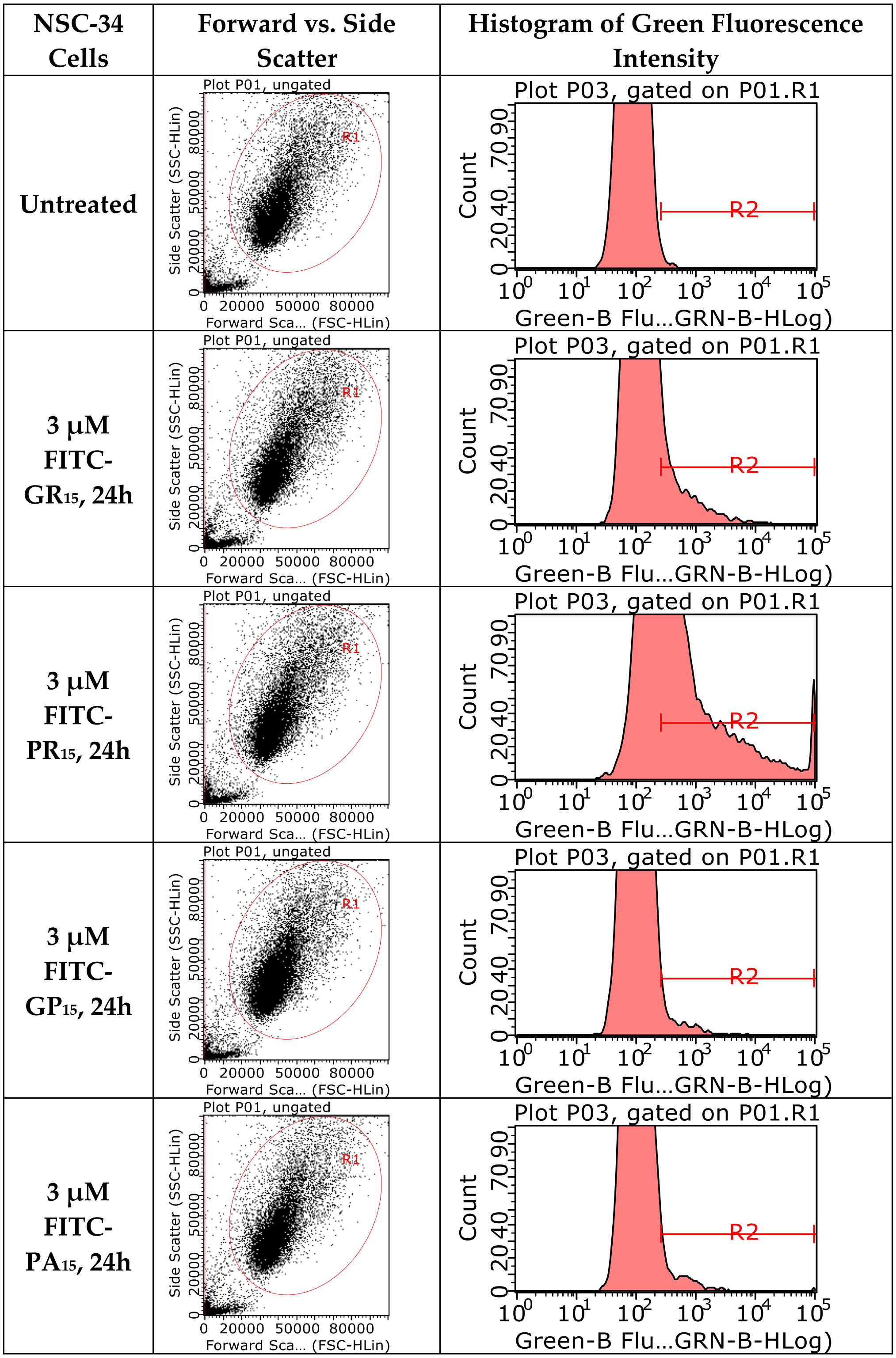

Supplementary Figure S2. Arginine-rich fluorescein isothiocyanate-labelled dipeptide repeat proteins (FITC-DRPs) remain in mouse spinal cord x neuroblastoma hybrid (NSC-34) cells after 24h incubation. Treatment conditions (Left), forward versus side scatter (Middle) and histograms of mean green fluorescence intensity (Right) indicate FITC-DRP internalization and remainder in NSC-34 cells over 24h, especially in the case of arginine-rich FITC-DRPs. Forward versus side scatter are gated to exclude cellular debris. Histograms are gated such that region R2 includes all green fluorescence signals more positive than untreated.

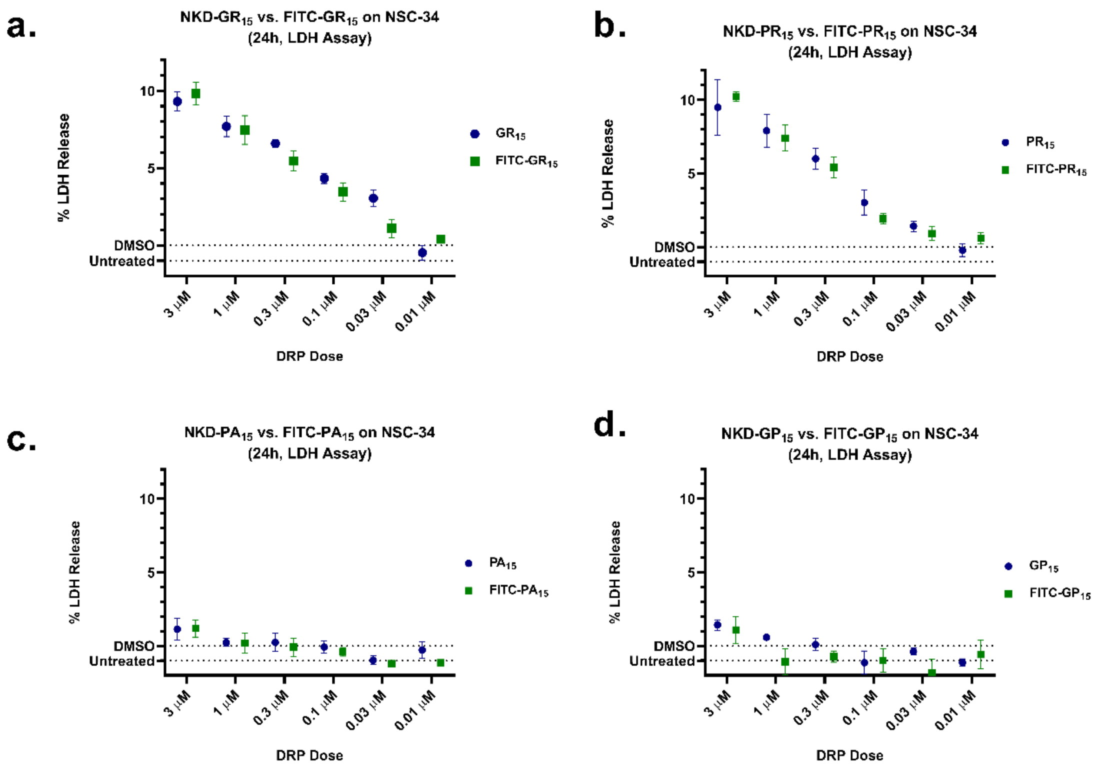

Supplementary Figure S3. 24h incubation of arginine-rich untagged (NKD) and fluorescein isothiocyanate-labelled (FITC) dipeptide repeat proteins (DRPs) on mouse spinal cord x neuroblastoma hybrid (NSC-34) cells increases lactate dehydrogenase (LDH) release in a significant, dose-dependent manner with no appreciable differences between NKD and FITC-DRP-induced LDH release. % LDH release was calculated based on absorbance (450 nm) values detecting the release of LDH from cells, where 0% LDH release reflects a negative control of untreated NSC-34 cells, and 100% LDH release reflects a positive control of lysed NSC-34 cells. Average % LDH release induced by dimethyl sulfoxide solvent (DMSO) and untreated cells are indicated by dotted lines.

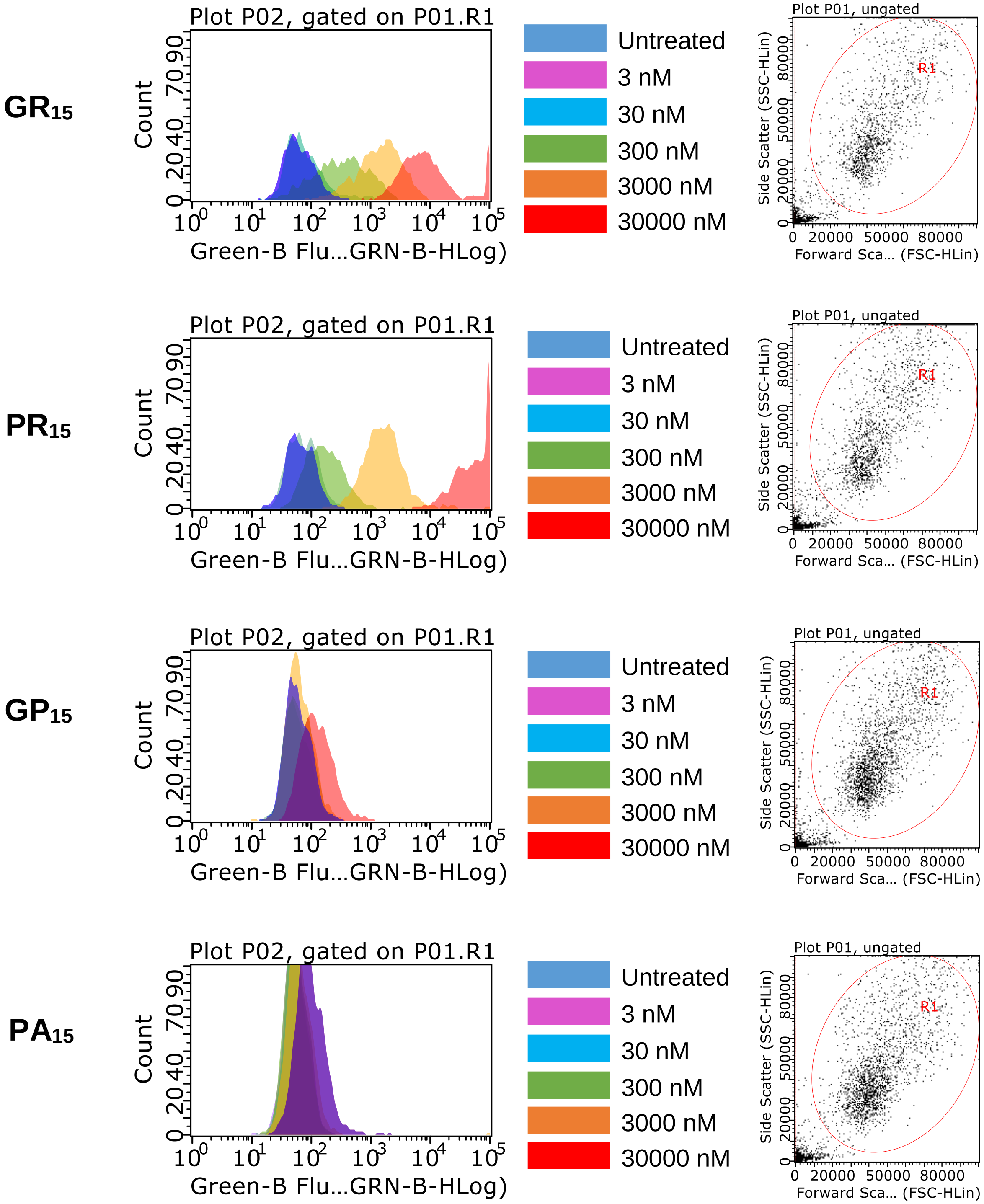

Supplementary Figure S6. Arginine-containing fluorescein isothiocyanate-labelled dipeptide repeat proteins (FITC-DRPs) are internalized by mouse spinal cord x neuroblastoma hybrid (NSC-34) cells in a rapid, dose-dependent manner. Uptake of DRPs after 1h incubation was assessed by increase in green fluorescence intensity as compared to untreated samples. Results in histograms are gated on R1, selecting for healthy cells and excluding debris, as depicted in forward versus side scatter plots (**right**).

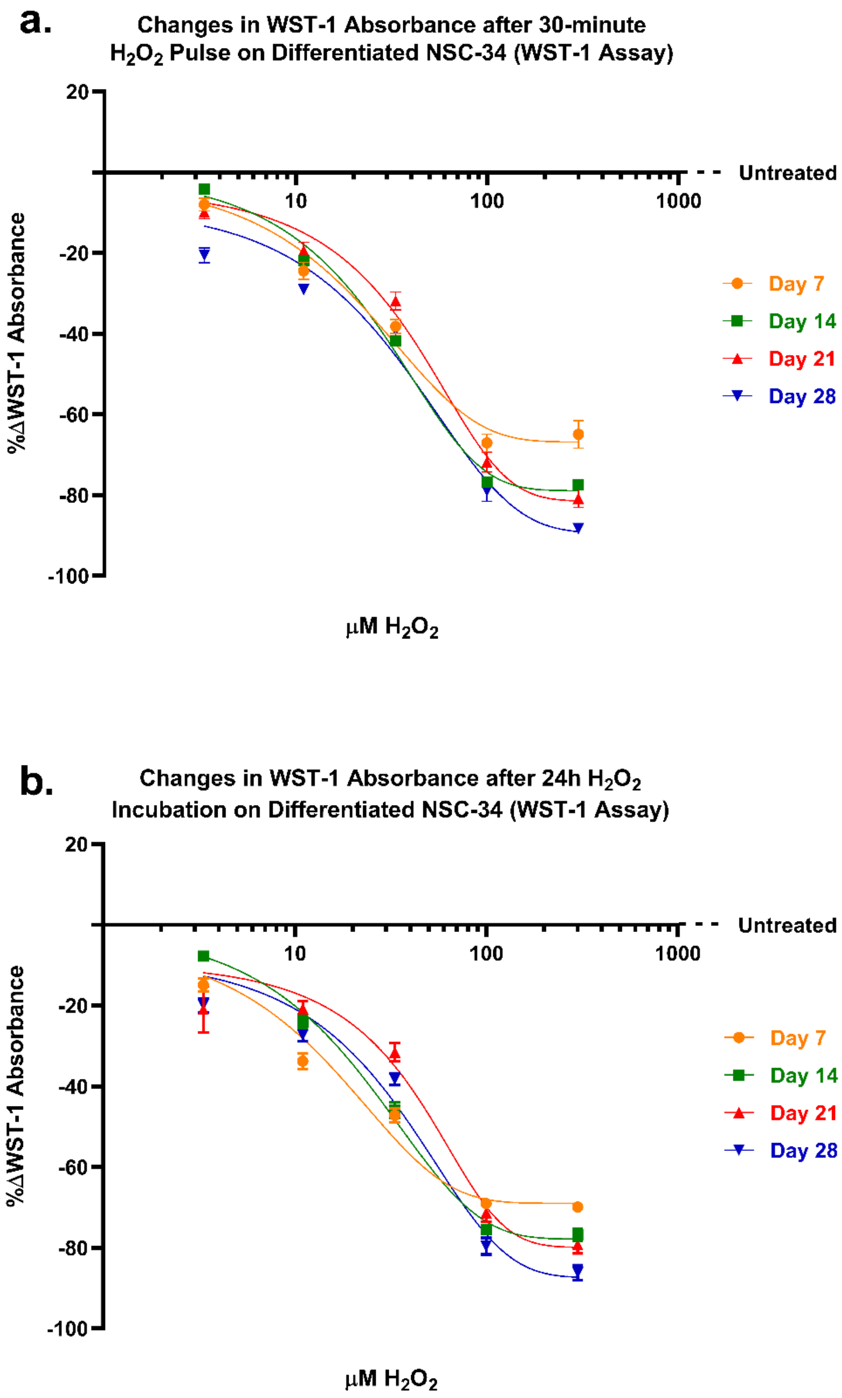

Supplementary Figure S7. WST-1 assay of mouse spinal cord x neuroblastoma hybrid (NSC-34) cells differentiated for either 7, 14, 21, or 28 days treated with either a 30-minute hydrogen peroxide pulse followed by 24h incubation (**a**), or 24h incubation with hydrogen peroxide (**b**), revealed no distinct differences in sensitivity based on differentiation state with the exception of the highest dose tested (300 µM).
